# Research on Long Jump Posture in School Physical Education Teaching Based on Video Analysis

**DOI:** 10.1155/2021/2324352

**Published:** 2021-11-16

**Authors:** Tianchang He, Danyang Lv, Zehao Li

**Affiliations:** ^1^Shenyang Sport University, Shenyang 110102, China; ^2^Shenyang Polytechnic College, Shenyang, China

## Abstract

Based on video, the human movement can be analyzed to achieve scientific training and skill improvement. Specifically, according to the video data during the movement, the human body can be detected and tracked, and relevant trajectory data can be obtained. On this basis, key motion parameters can be obtained and quantitative analysis of motion can be achieved. This paper uses video processing technologies to analyze the long jump posture in physical education. According to the video sequences measured during the athlete's long jump, the target detection and tracking algorithms are used to obtain the athlete's trajectory after preprocessing. Afterwards, further processing is carried out to calculate speed, angle, posture, and other related information to assist scientific sports training. The experimental results based on the measured data show that the algorithm can realize the analysis of the long jump scene and complete the quantitative analysis of the key indicators of the athletes. The research results can effectively support school physical education and guidance training and also provide a reference for other competitive video analysis.

## 1. Introduction

Human motion analysis has received much attention in the field of computer vision in recent years [1–3]. It is an important technology that combines contemporary biomechanics and computer vision. It has a very broad and important application in motion analysis, virtual reality, and relevant fields. Competitive sports videos capture the various actions of athletes during training or competitions with cameras. Digital video processing and image analysis techniques can be used to analyze and compare the action status and details, allowing athletes to intuitively understand their own shortcomings and advantages in order to enhance the level of sports training [4–7]. Video research based on moving backgrounds can overcome the disadvantages such as fixed scenes and small observation ranges brought by stationary cameras, thus realizing real-time tracking and expand observation ranges. It has been widely used in military and civil affairs such as human-computer interaction, image navigation, and battlefield surveying, which have achieved rich results. The U.S. Department of Defense launched the Visual Surveillance and Monitoring (VSAM) research project and realized the functions of battlefield situation analysis, refugee management, and key site control [8–10]. In the European Union (EU), French and British research institutions jointly developed a set of tools to improve the public transportation network management system [11–13]. In the field of sports science research, video technology is used to automatically segment the complex technical movements in the training process of athletes and the postures in dance training for recognition and guidance training [14–16]. In China, the video analysis research studies have been analyzed for different kinds of sports, and good results have been obtained.

In this paper, the video processing technologies are used in long jump video analysis to calculate the key parameters of athletes in the process of long jump, so as to provide support for targeted improvement of training methods. First, the athlete's long jump video is processed to improve the image quality of each frame [17–20]. On this basis, the video target detection technology is used to obtain the athlete in the scene as the object to be analyzed. Then, the video tracking technology is used to continuously track the athlete's trajectory during the long jump, so as to realize the record of the whole process [21–23]. Finally, according to the athlete's trajectory, by analyzing the relative relationship between the video frames, the relationship information of the athlete's speed, posture angle, etc., at each moment can be calculated [24, 25]. Based on the obtained parameters, the athlete's long jump actions and habits can be studied and judged, so as to provide support for physical education and targeted training. In the experiment, the proposed method is tested and verified by using the measured data from the sports meetings. The experimental results show that the proposed method can effectively analyze the athlete's posture in the long jump and has a supporting role for scientific sports training and teaching.

## 2. Methodology

**Figure 1:**
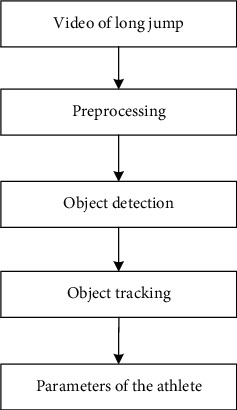
Basic process of the proposed method.

Competitive sports are generally more intense, with a short completion time usually less than 20 seconds. It is difficult for coaches to intuitively judge athletes' sport indicators in a short period of time. In order to help the athletes achieve excellent results in the competition, daily training is essential [15–17]. With the development of science and technology, coaches can make full use of various modern technologies in the training. During the training or competition, by collecting the athlete's hitting action and tracking the ball, it can be effectively judged whether the action is standard and effective, so as to help the athlete to correct it in time. It can improve the level of sports skills. This paper applies the video processing technology to the analysis of long jump movement. The basic idea can be described as Figure 1. It mainly distinguishes three stages: video preprocessing, video target detection, and target tracking, to achieve the closed-loop monitoring of long jump athletes. And, based on the results, the key parameters can be analyzed and calculated.

### 2.1. Preprocessing

Video monitoring of movement and ball movement trajectory plays an important role in the athlete training and predicting the ball's fall point. Video surveillance runs over time, so the collected video images have a certain time sequence, which is called a sphere video sequence image [18–20]. Since video surveillance works in an outdoor environment, it will be affected by the external environment. And, the quality of the collected images may not be high. Therefore, before tracking the sphere motion trajectory, the target video sequence image needs to be preprocessed, mainly including image grayscale. There are three types of processing technologies: image denoising, image denoising, and image enhancement. Among them, image graying and image denoising are more mature. This paper focuses on image enhancement based on homomorphic filtering.

After the image is denoised, it will inevitably cause a part of the image details to be blurred. In addition, the collected image will be affected by a different illumination, and the brightness will be uneven. The above two factors will reduce the contrast of the image and affect the detection of the spherical target in the image [21]. To this end, the image enhancement processing is required. Homomorphic filtering is a special method for image contrast enhancement and compression of image brightness range in the frequency domain [22]. The specific operation process is as follows.

Step 1: assuming that the original image *f*(*x*, *y*) is composed of the product of illuminance and reflection, the formula is as follows:
  (1) $$\begin{array}{c} f\left(x,y\right)=a\left(x,y\right)\cdot b\left(x,y\right), \end{array}$$
where *a*(*x*, *y*) is the illuminance and *b*(*x*, *y*) is the reflection component.
Step 2: equation (2) is used to perform logarithmic transformation on both sides of equation (1) to separate illuminance and reflection:
  (2) $$\begin{array}{c} \ln\left(f\left(x,y\right)\right)=\ln\left(a\left(x,y\right)\right)+\ln\left(b\left(x,y\right)\right). \end{array}$$
Step 3: the Fourier transform is performed based on equation (3), from time domain to frequency domain:
  (3) $$\begin{array}{c} F\left(u,v\right)=A\left(u,v\right)+B\left(u,v\right), \end{array}$$
where *A*(*u*, *v*) and *B*(*u*, *v*) are the Fourier transforms of ln(*a*(*x*, *y*)) and ln(*b*(*x*, *y*)), respectively.
Step 4: the filter processing *F*(*u*, *v*) is used with a transfer function of *H*(*u*, *v*) to obtain
  (4) $$\begin{array}{c} Y\left(u,v\right)=H\left(u,v\right)F\left(u,v\right)\\ =H\left(u,v\right)A\left(u,v\right)+H\left(u,v\right)B\left(u,v\right), \end{array}$$
where
  (5) $$\begin{array}{c} H\left(u,v\right)=\left(r_{H}-r_{L}\right)\left[1-\exp\left(-c\times \frac{D^{2}\left(u,v\right)}{D_{0}^{2}}\right)\right]+r_{L}, \end{array}$$
  (6) $$\begin{array}{c} D\left(u,v\right)=\left[{\left(u-\frac{M}{2}\right)}^{2}+{\left(v-\frac{N}{2}\right)}^{2}\right]. \end{array}$$
In the above equations, *r*_*L*_ and *r*_*H*_ are the adjustment parameter (*r*_*L*_ <1, *r*_*H*_ >1); *c* is a constant, which controls the sharpness of the filter function, here it is 0.5, *D*(*u*, *v*) is the distance between the point (*u*, *v*) and the center frequency point (*M*, *N*), and *D*_0_ is the variance of the Gaussian function.
Step 5: the inverse Fourier transform is performed on the filtered image to convert it from frequency domain to time domain:
  (7) $$\begin{array}{c} z\left(x,y\right)=\xi^{-1}\left\{Y\left(u,v\right)\right\}=a^{\prime }\left(x,y\right)+b^{\prime }\left(x,y\right), \end{array}$$
where
  (8) $$\begin{array}{c} a^{\prime }\left(x,y\right)=\xi^{-1}\left\{H\left(u,v\right)A\left(u,v\right)\right\}, \end{array}$$
  (9) $$\begin{array}{c} b^{\prime }\left(x,y\right)=\xi^{-1}\left\{H\left(u,v\right)B\left(u,v\right)\right\}. \end{array}$$
Step 6: the exponential operation is performed on *z*(*x*, *y*) to obtain an enhanced image:
  (10) $$\begin{array}{c} g\left(x,y\right)=e^{z\left(x,y\right)}=e^{a^{\prime }\left(x,y\right)}e^{b^{\prime }\left(x,y\right)}. \end{array}$$

### 2.2. Detection of Moving Objects

The moving target detection refers to the extraction of moving actions and spherical targets from the entire collected image, which is the key to subsequent moving target tracking [16–18]. At present, there are mainly three methods for target detection, namely, the frame difference method, the background subtraction method, and the optical flow method. In this section, the background subtraction method is chosen and employed. The background difference method is a preacquisition of a background image template, and then, the real-time collected video image containing the sphere and the background image template are differentiated to realize the separation of the moving object area from the scene. Define *Z*(*x*, *y*, *t*) as the image at the time *t* in the video image sequence, and the background image is *U*(*x*, *y*, *t*) at the time *t*; then, the difference image at the time *t* is

(11) $$\begin{array}{c} d\left(x,y,t\right)=\left|Z\left(x,y,t\right)-U\left(x,y,t\right)\right|. \end{array}$$

The threshold judgment is performed on the difference image *d*(*x*, *y*, *t*) at the time *t*. When it is greater than the threshold *T*, it is considered that there is a moving target in the monitored scene. Otherwise, it is considered that there is no moving target. Theoretically, as long as the background does not change, the background difference method can achieve a good target detection effect. However, once the background image template has subtle changes, such as changes in light, water patterns, and leaf shaking, the contrast effect of the background image template will be used. Therefore, the Gaussian model is used to improve the background difference method, that is, a Gaussian model is established for each point in each background image. And, several Gaussian models are used to jointly describe the background pixels so that the background model can be continuously updated. The background model can respond to changes brought about by factors such as changes in illumination, water patterns, and leaf shaking in real time.

### 2.3. Tracking of Moving Objects

The motion trajectory tracking refers to the process of finding various positions belonging to the same moving target in a video image sequence and connecting the positions of various points into a running line [14–22]. The motion trajectory tracking is mainly divided into two stages of tasks, namely, moving target marking and moving target feature matching tracking.

#### 2.3.1. Moving Target Marking

If the abovementioned processing and detection process are repeated for each frame of image, the amount of calculation is relatively large. Therefore, after the moving sphere target detection of the initial image is completed, some invariant or partially invariant characteristics of the sphere can be extracted, such as objects. The centroid, color, size, area, texture, etc. of the moving target are marked with a matching function. Then, these features are directly used to match and recognize the next frame of image through a matching function, and the position of the moving target in the video image can be clarified.

#### 2.3.2. Feature Matching and Tracking of Moving Target

After selecting the features of the moving object, the feature matching is performed in different frames to achieve moving target tracking. The target tracking is implemented here by choosing the Kalman filter method. The basic principle is as follows. First, a Kalman filter is designed for motion measurement, and then, it is used to predict the position of the sphere in the next frame of image. Then, the approximate search area is divided, and the matching algorithm is used to match the features of the moving target in the area. When all the features are matched, it is considered that the moving target is within the range, and the corresponding target position is marked. And, the recursive detection is continued according to this law to realize the continuous tracking of the moving target [22].

## 3. Analysis of Key Indexes

The long jump event is a sport that consists of three consecutive jumps, consisting of hop, skip, and jump, after the athlete has accelerated the approach. The relevant key technical indicators include approach speed, speed utilization, pedal accuracy, triple jump ratio, and movement angle value. This paper focuses on quantitative analysis of approach speed and angle of motion. In the experiment, the relevant video data was collected from the sports meetings.

### 3.1. Approach Speed Analysis

Approach speed is an important parameter that affects long jump performance. According to the rules of the triple jump venue, the wind gauge is 20 meters away from the starting board. Take the wind gauge and the take-off board as signs, and set the distance between the two as *S*; when the athlete arrives, enter the set rectangular effective range and start to calculate the number of video frames. The used time is obtained as *T*. Accordingly, the average speed *V* during this period can be calculated as *V*=*S*/*T*.

In the specific experiment, the long jump data of athletes in a sports meeting is selected, and the video playback rate is 1020 frames/min. The number of frames corresponding to the athletes entering the effective rectangular range of the wind gauge and the take-off board is the 3rd frame and the 38th frame, respectively. According to the above formula, the average speed of the athlete is about 9.532 m/s.

### 3.2. Angle Analysis

**Figure 2:**
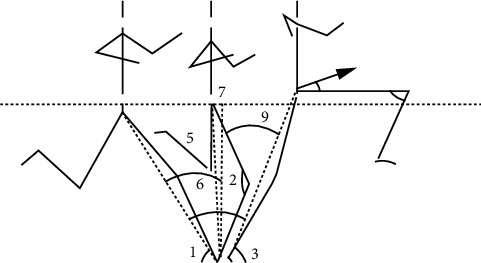
Illustration of key angles in long jump.

Body posture affects the final result of long jump. As shown in Figure 2, the key actions in the long jump process are analyzed. The movement process is mainly determined by three angles: the ground clearance angle (∠1), the take-off angle (∠3), and the knee joint angle (∠2 and ∠4). These three angles can quantitatively measure the take-off and landing postures of the movement and reflect the way of exerting force and the level of strength of the movement. Therefore, through the analysis of the above angles, the related habits of remote mobilization can be effectively studied and judged, and scientific training can be guided on this basis. In order to analyze the above angle information in a refined manner, the entire process of the athlete's long jump can be video-monitored and processed afterwards.

**Figure 3:**
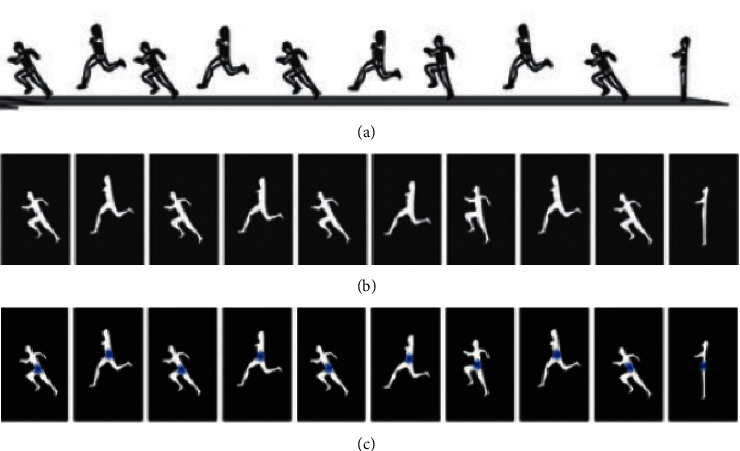
Analysis of key angles in long jump. (a) Illustration of the behaviors during long jump. (b) Segmentation results of the objects. (c) Illustration of feature extraction.

When calculating the angle index, it is necessary to perform edge detection, contour extraction, contour moment calculation, corner detection, and other features on the athlete's image.  Figure 3 shows a series of actions of the athlete during the long jump, and the result after the completion of the video target detection, tracking, and feature extraction. On this basis, the key angles in the long jump process can be analyzed and calculated.

Based on the completion of video detection and tracking, the algorithm ideas for calculating the ground clearance angle (take-off angle) are as follows:

  Step 1: after separating the moving target, the target contour is extracted. In the experiment, the Sobel edge detection operator is used for contour extraction;
  Step 2: according to the moment characteristics of the contour, the athlete's center of mass is extracted and the coordinates of the touchdown point can be obtained. The contour moment is a statistical feature obtained by integrating all the pixels on the contour.
  Step 3: the landing angle can be calculated according to the triangle cosine formula.

In the experiment, the actual measurement video of a certain sports meeting was used to analyze the take-off angle. The coordinates of the center of gravity of the remote mobilization are (125, 76), the coordinates of the landing location are (163, 148), and the calculated take-off angle is 61.2°.

Similarly, according to the results of video detection and tracking, the algorithm of calculating the knee joint angle is as follows:

  Step 1: the contour processing is performed on the target moving image.
  Step 2: the small interference contours are eliminated according to the contour area and other characteristics, and we finally get the athlete contour.
  Step 3: the corners of the contour image are detected and the corners with numbers are marked. In the experiment, the Harris operator is used to detect the corner points.
  Step 4: the corner point number of interest is entered in order to accurately determine the position of the detected corner point.
  Step 5: a circle with the corner is used as the center and the intersection with the contour to form a triangle.

## 4. Conclusion

Aiming at the long jump teaching problem, this paper uses video processing technology to analyze the athlete's posture and optimizes and improves the teaching and training methods based on the key parameters of the measurement. For the athlete's long jump video, the whole process of the athlete is tracked through preprocessing, target detection, and target tracking, and the relevant movement trajectory is obtained. On this basis, the speed, angle, and other related information of the athletes during the long jump can be calculated according to the relative relationship between the image sequences. At the same time, the athlete's body area can be segmented at each moment so that its posture can be further analyzed. The research results of this paper can be used for efficient physical education and targeted training of athletes. In the follow-up research, further analysis and processing will be done on the athlete's posture and other related information at each moment of the video to enhance the auxiliary effect for the long jump movement.

## Data Availability

The dataset can be accessed upon request.
